# Characterization of hydroxypropyl-beta-cyclodextrins used in the treatment of Niemann-Pick Disease type C1

**DOI:** 10.1371/journal.pone.0175478

**Published:** 2017-04-17

**Authors:** Alfred L. Yergey, Paul S. Blank, Stephanie M. Cologna, Peter S. Backlund, Forbes D. Porter, Allan J. Darling

**Affiliations:** 1 Eunice Kennedy Shriver National Institute of Child Health and Human Development, National Institutes of Health, Bethesda, MD, United States of America; 2 Department of Chemistry, University of Illinois at Chicago, Chicago, IL, United States of America; 3 Vtesse, Inc., Gaithersburg, MD, United States of America; Steno Diabetes Center, DENMARK

## Abstract

2-Hydroxypropyl-beta-cyclodextrin (HPβCD) has gained recent attention as a potential therapeutic intervention in the treatment of the rare autosomal-recessive, neurodegenerative lysosomal storage disorder Niemann-Pick Disease Type C1 (NPC1). Notably, HPβCD formulations are not comprised of a single molecular species, but instead are complex mixtures of species with differing degrees of hydroxypropylation of the cyclodextrin ring. The degree of substitution is a critical aspect of the complex mixture as it influences binding to other molecules and thus could potentially modulate biological effects. VTS-270 (Kleptose HPB) and Trappsol^®^ Cyclo^™^ are HPβCD products under investigation as novel treatments for NPC1. The purpose of the present work is to compare these two different products; analyses were based on ion distribution and abundance profiles using mass spectrometry methodology as a means for assessing key molecular distinctions between products. The method incorporated electrospray ionization and analysis with a linear low-field ion mobility quadrupole time-of-flight instrument. We observed that the number of hydroxypropyl groups (the degrees of substitution) are substantially different between the two products and greater in Trappsol Cyclo than in VTS-270. The principal ions of both samples are ammonium adducts. Isotope clusters for each of the major ions show doubly charged homodimers of the ammonium adducts. In addition, both products show doubly charged homodimers from adduction of both a proton and ammonium. Doubly charged heterodimers are also present, but are more intense in Trappsol Cyclo than in VTS-270. Based on the analytical differences observed between VTS-270 and Trappsol Cyclo with respect to the degree of substitution, the composition and fingerprint of the complex mixture, and the impurity profiles, these products cannot be considered to be the same; the potential biological and clinical implications of these differences are not presently known.

## Introduction

Cyclodextrins are cyclic oligosaccharides that, for many years, have been used to modulate the composition of cholesterol and other lipids in biological membranes and have also been used as pharmaceutical excipients in the formulation of hydrophobic drugs [1, 2]. More recently, 2-Hydroxypropyl-β-cyclodextrins (HPβCDs) have gained attention as a potential therapeutic intervention for Niemann-Pick Disease Type C1 (NPC1), an autosomal-recessive, rare, fatally progressive, neurodegenerative lysosomal storage disease characterized by endo-lysosomal accumulation of cholesterol and other lipids [3–7]. Currently, there are no approved pharmacological drugs for the treatment of NPC1 in the United States, and options available to patients are limited to supportive therapies and use of miglustat, which is off-label in the United States; miglustat is an iminosugar that reduces glycosphingolipid production through the inhibition of the enzyme glucosylceramide synthase [8]. Management of the disease is mainly aimed at symptomatic relief [9, 10], leaving unmet medical needs for NPC1 patients. However, progress has been made recently in the development of novel therapeutics for NPC1 [4, 11, 12]. In the United States and European Union, two HPβCD products, VTS-270 (Vtesse, Inc., Gaithersburg, MD) and Trappsol^®^ Cyclo^™^ (CTD Holdings, Inc., Alachua, FL) have received orphan drug designations. VTS-270 uses Kleptose^®^ HPB (Roquette Pharma, France) as the active ingredient, which has shown highly encouraging results in a phase 1/2a clinical trial (NCT01747135) and is currently being studied in a global pivotal phase 2b/3 clinical trial (NCT02534844) as a treatment for the neurological manifestations of NPC1 [7, 13]. Trappsol Cyclo is a second HPβCD and is the subject of a recent investigational new drug filing. Given the use of these HPβCD products in NPC1 clinical investigations, it is important to understand if they are chemically different and clinically equivalent. Not only has there been extensive interest in HPβCD materials for NPC1, but also for a number of other disorders including atherosclerosis, Alzheimer’s disease, Parkinson’s disease, and Huntington’s disease [14].

**Fig 1:**
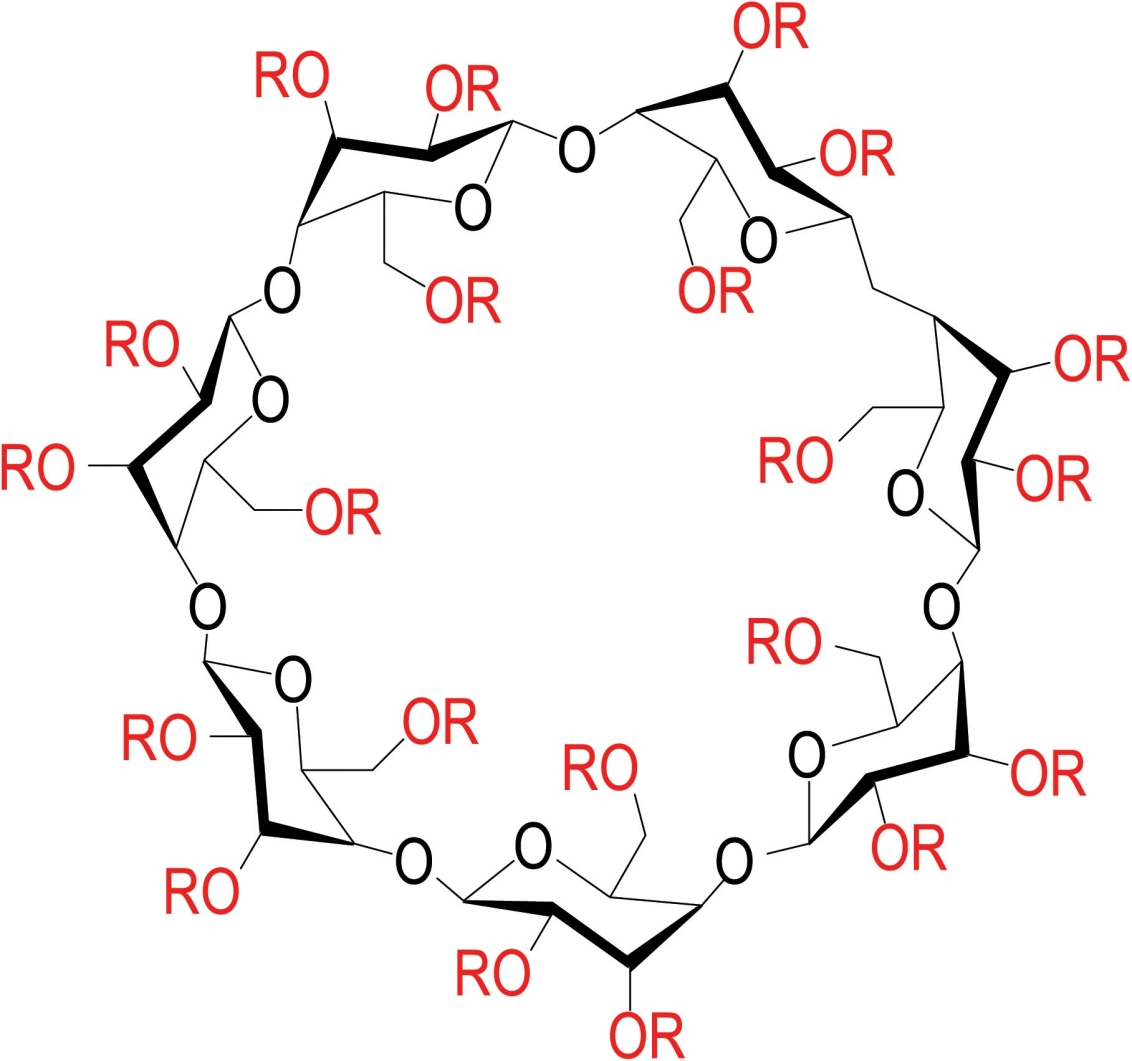
Structure of 2-hydroxypropyl-β-cyclodextrin (HPβCD) where R = H or CH_2_-CHOH-CH_3_. There are 21 (red) hydroxyl groups on βCD which are potential substitution sites for the condensation reaction with propylene oxide to yield various species of HPβCD with different degrees of substitution depending upon how many sites are substituted.

Unlike most FDA-approved drugs, which are characterized as single chemical or molecular species, HPβCDs are complex mixtures of different species, and variations in production may lead to differences in composition. HPβCDs are synthesized by condensation between β-cyclodextrin (βCD) and propylene oxide [15]. There are 21 hydroxyl groups on βCD, all of which are potential sites for the condensation reaction (Fig 1) [15]. Following condensation, the number of substituted hydroxypropyl groups on the cyclodextrin ring varies with synthesis conditions and leads to a distribution in the degrees of substitution on HPβCD [15]. Importantly, the condensation reactions do not lead to a single molecular HPβCD species but rather to a complex mixture of HPβCD species with differing degrees of hydroxypropylation. The average degree of substitution (DS) is a critical characteristic of the complex mixture, as it influences the ability of HPβCDs to bind other molecules [16] and affects the degree of aqueous solubility [1], which may influence biological activity. Thus, in the setting where these materials are used as therapeutic agents, the need to accurately characterize the complex mixture of HPβCD species becomes necessary in order to attain consistent clinical safety and efficacy responses. While quality standards for HPβCDs used as pharmaceutical excipients are based on United States Pharmacopeia (USP) specifications [17], these characterization criteria were not developed for active therapeutic agents that directly treat disease and are considered to be unsatisfactorily broad and insufficiently specific for such applications. For potential therapeutic applications, it is important to characterize HPβCD mixtures to aid in the future determination of which specific components may be therapeutically active and which may be toxic. Herein, we report an approach to characterize HPβCDs using ion mobility mass spectrometry to determine both the DS and describe specific ions arising from interactions between HPβCDs that yield homo- and heterodimeric ions of both protonated and ammonium adducts; we term these characteristic interaction ions a “molecular fingerprint.” Using this method, VTS-270 and Trappsol Cyclo were analyzed and characterized, and substantial chemical differences were observed.

## Materials and methods

### Materials

All reagents were used as supplied unless otherwise noted. Water was purified using a Hydroservices system (Levittown, PA) and then polished using a Millipore Simplicity UV purification unit (Billerica, MA); HPLC grade acetonitrile (ACN) was obtained from Burdick and Jackson (Morris Plains, NJ).

VTS-270 (Vtesse, Inc., Gaithersburg, MD) was supplied in 14 separate synthetic batches for comparison. Two separate synthetic batches of Trappsol Cyclo (CTD Holdings, Inc., Alachua, FL) were used for comparison. HPβCD solutions were prepared as a 1 mg/mL stock in water and infused at either a 200-fold dilution in 1:1 water:ACN or a 400-fold dilution in water:80% (v/v) ACN. Based on the chemical structure and stock solution, a 200-fold dilution represents a concentration of approximately 5 μM.

### Measurement approach

An Agilent Model 6560 Ion Mobility Quadrupole Time-of-Flight Mass Spectrometer (Agilent Technologies, Santa Clara, CA) was used for all measurements. The instrument has been described previously [18]. In brief, electrospray-generated ions were formed by direct infusion of HPβCD solutions using a nano-electrospray ionization (ESI) source with a flow rate set at 600 nL/min, V_cap_ potential of 1500 V, and sheath gas flow at 5.0 L/min at 150^°^C. With this instrument, ions are sampled into the drift tube through a series of ion funnels, the last of which serves as a trapping ion funnel to deliver ions at 80-msec intervals to the ion mobility drift tube. RF amplitudes in the trapping ion funnel were adjusted to optimize ion intensities. The drift tube was operated with a N_2_ bath gas pressure at 3.94 Torr and 27^°^C as well as 1450 V potential across the tube. Upon exiting the drift tube, ions passed through another series of optics and were mass analyzed by a high-resolution Q-TOF mass spectrometer. Ion signals were collected for 3-minute intervals prior to analysis and measurements were made in duplicate each day for a total of 3 days over 3 weeks (ie, 12 replicate measurements). All spectra collected were analyzed using Agilent Mass Hunter software (version 7.01) for visualization and manually interpreted and annotated. Mass assignments were based upon accurate mass measurement. Ion collision cross sections were determined using software supplied in this same package.

### Ion collision cross section determination

Under conditions of low drift fields in an appropriate pressure regimen, as defined by Mason and McDaniel [19] and approximating both an ion and the buffer gas as hard spheres, the collision cross section, Ω, can be calculated in units of Å^2^ from the expression:

$$\Omega =\frac{{\left(18\pi \right)}^{1/2}}{16}\frac{ze}{{\left(k_{B}T\right)}^{1/2}}{\left[\frac{1}{m_{i}}+\frac{1}{m_{B}}\right]}^{1/2}\frac{1}{K_{0}}\frac{1}{N^{*}}$$

where k_B_ is the Boltzmann constant, z and e are the charge state and electronic charge, respectively, N^*^ is the gas number density in the drift tube, m_i_ is the ion mass, and m_B_ is the mass of the buffer gas. Widespread usage has allowed the spherical assumptions to be extended to what are clearly non-spherical ions and to non-spherical, polarizable drift gases. K_0_ is determined from measurements of apparent ion drift times that are corrected for passage time in the instrument outside of the drift tube region and transformed to STP conditions.

## Results

All batches of VTS-270 and both Trappsol Cyclo batches showed consistent behavior; data are shown for a representative single batch for each product.

### Ion mobility-mass spectrometry profiles of VTS-270 and Trappsol Cyclo

**Fig 2:**
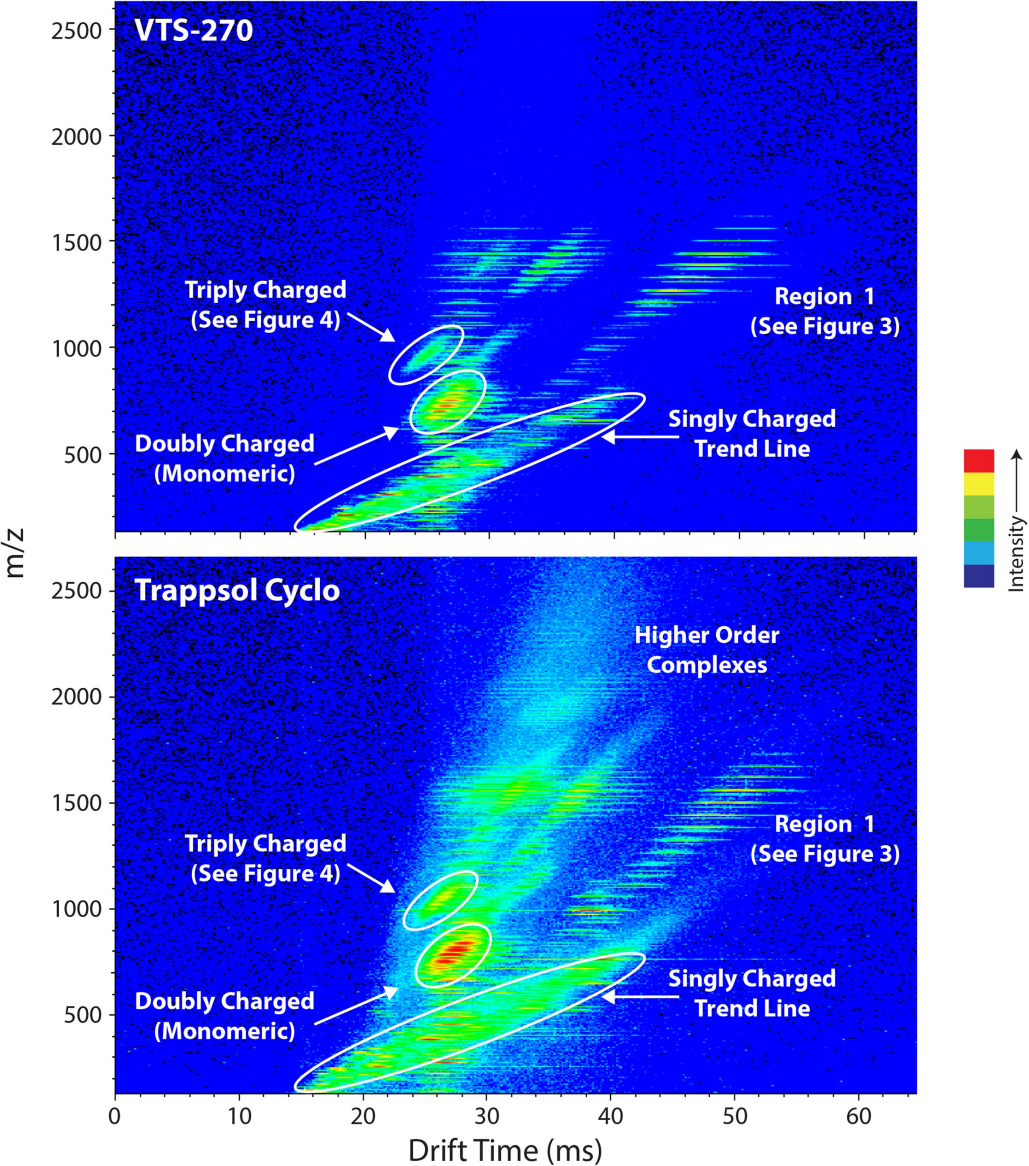
Ion mobility false color representations (“heat map”) of VTS-270 and Trappsol Cyclo. Ion mobility drift time (msec) is shown on the x axis and m/z on the y axis. Heat map intensities are shown with colors ranging from lowest intensity in blue to highest intensity in red. Materials were infused under the same preparation conditions and at the same concentration of HPβCD. Trappsol Cyclo exhibits greater chemical heterogeneity than VTS-270 as observed by the more complex overall heat map and the m/z singly charged trend line (long oval-shaped area), the region labeled “higher order complexes,” and the region corresponding to triply charged dimers.

Initial experiments compared the ion mobility profile of the two HPβCD products. Fig 2 shows “heat map” false color representations of the instrument response in ion mobility-space of VTS-270 (upper panel) and Trappsol Cyclo (lower panel). Ions that have larger three-dimensional structures have longer drift times than ions with smaller structures [20]. Ions with the same m/z values but different charge states have different drift times, with higher charge state ions having shorter drift times [20]. These components of the ion mobility response give rise to the several “families” of ion responses corresponding to ions with related structures and different charge states seen in the two panels of Fig 2. Considering that the two materials are infused under the same preparation conditions and at the same concentration, it is apparent that there is a much greater level of non-specific chemical “noise” associated with Trappsol Cyclo compared with VTS-270, particularly in the lower m/z regions over drift times up to approximately 45 msec. In addition, the Trappsol Cyclo heat map shows the presence of ions at m/z >2000 and drift times between 30 and 40 msec, which are not observed in VTS-270. These species correspond to aggregates of multiple HPβCDs at high charge states.

### Mass spectra of VTS-270 and Trappsol Cyclo

**Fig 3:**
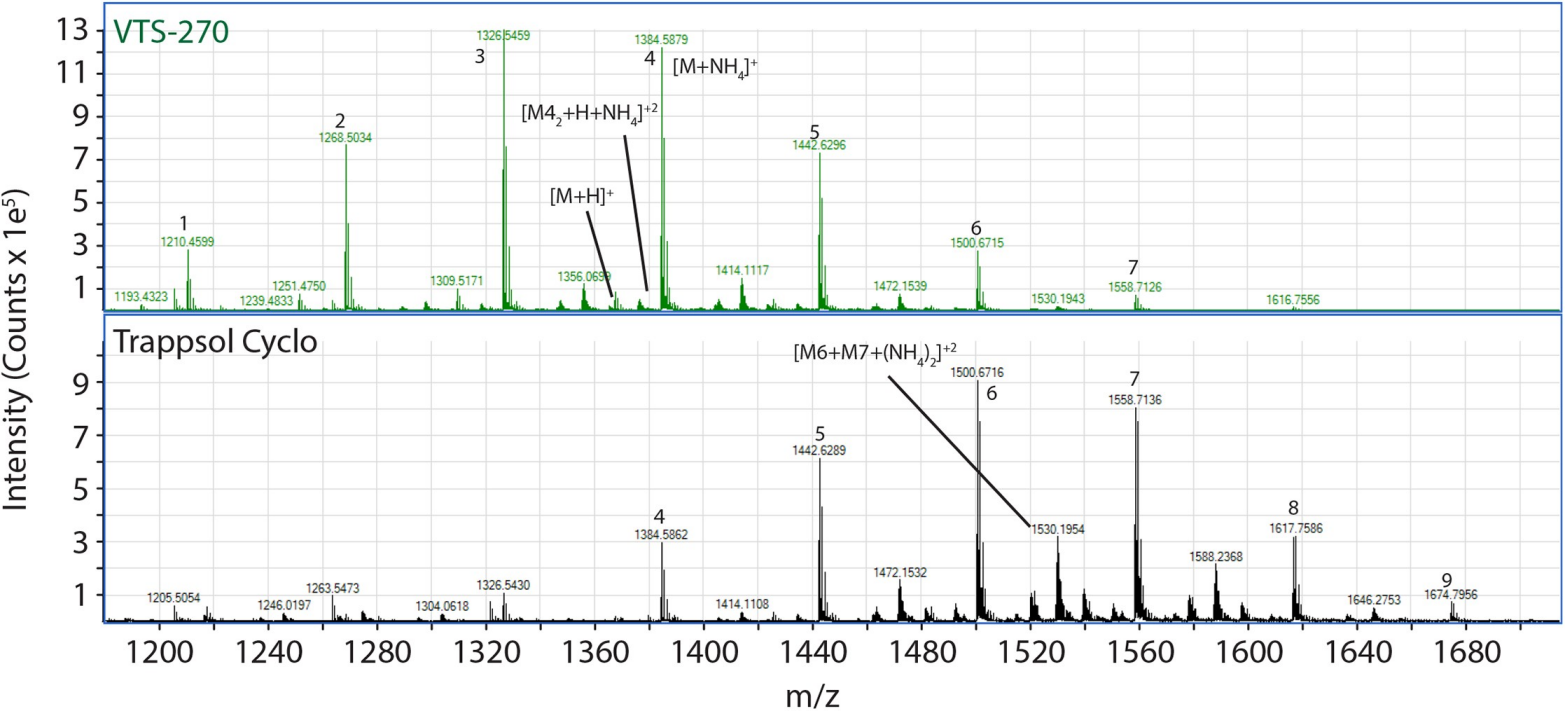
Mass spectra of a portion of the heat maps of VTS-270 and Trappsol Cyclo showing the m/z region dominated by singly charged NH4+ adduct ions. The spectra represent the areas in Fig 1 that are labeled “Region 1.” See text for detailed description of the spectra. The numbers above the most intense ions of each spectrum show the degrees of substitution of the ammonium adduct for that species (eg, nominal mass 1384, corresponding to the ammonium adduct of a degree of substitution equal to 4). The spectra show clear differences between VTS-270 and Trappsol Cyclo in the distributions of ion intensities corresponding to the degrees of substitution, with Trappsol Cyclo having a greater range and higher degrees of substitution.

Additional differences between VTS-270 and Trappsol Cyclo can be seen in Fig 3, which presents the mass spectra extracted from Region 1 denoted on Fig 2. These mass spectra exhibit resolutions of 20,000 or greater and peak assignments are consistent with 10 ppm or better mass accuracy when compared with expected values. This region of the heat maps covers the mass range 1200 to 1700 Da and drift times between 25 and 50 msec. Fig 3 represents a “compression” of all the ion intensities in this drift region and therefore combines the intensities of multiply charged ions of the same m/z, namely, monomeric singly charged and doubly charged ions formed from two HPβCD molecules as either two molecules of a single DS (homodimers) or two different degrees of substitution (heterodimers). The most intense ions in the spectra are those formed by NH_4_^+^ adduction to neutral HPβCD species with smaller contributions from protonated adducts. The spectra show a distribution of ion intensities corresponding to the degrees of substitution of the two materials studied, and demonstrate clear differences between them. The numbers above the most intense ions of each spectrum show the DS of the ammonium adduct for that species (eg, nominal mass 1384 corresponds to the ammonium adduct of a DS = 4). Close examination of the isotope clusters for each of the major ions shows both doubly charged homodimers of ammonium adducts (ie, [DS_n_ + NH_4_]_2_^+2^ of the ammonium adducts). In the case of Trappsol Cyclo, doubly charged dimers of the protonated ions are observed. Both materials show doubly charged homodimers from adduction of both a proton and ammonium; one of these is noted in the upper panel of Fig 3. Doubly charged heterodimers ([DS_n_ + DS_n-1_ + 2NH_4_]^+2^ are noted for the case of degrees of substitution of 6 to 7 at nominal mass 1530) are formed in both materials but are far more intense in Trappsol Cyclo than in VTS-270. Finally, we determined that all of these differences were maintained at ~2.5 μM concentrations (400-fold dilution) in 80% ACN, indicating that these dimers have strong intermolecular associations and that this does not suggest a concentration dependence in ion formation at the working concentrations studied.

**Fig 4:**
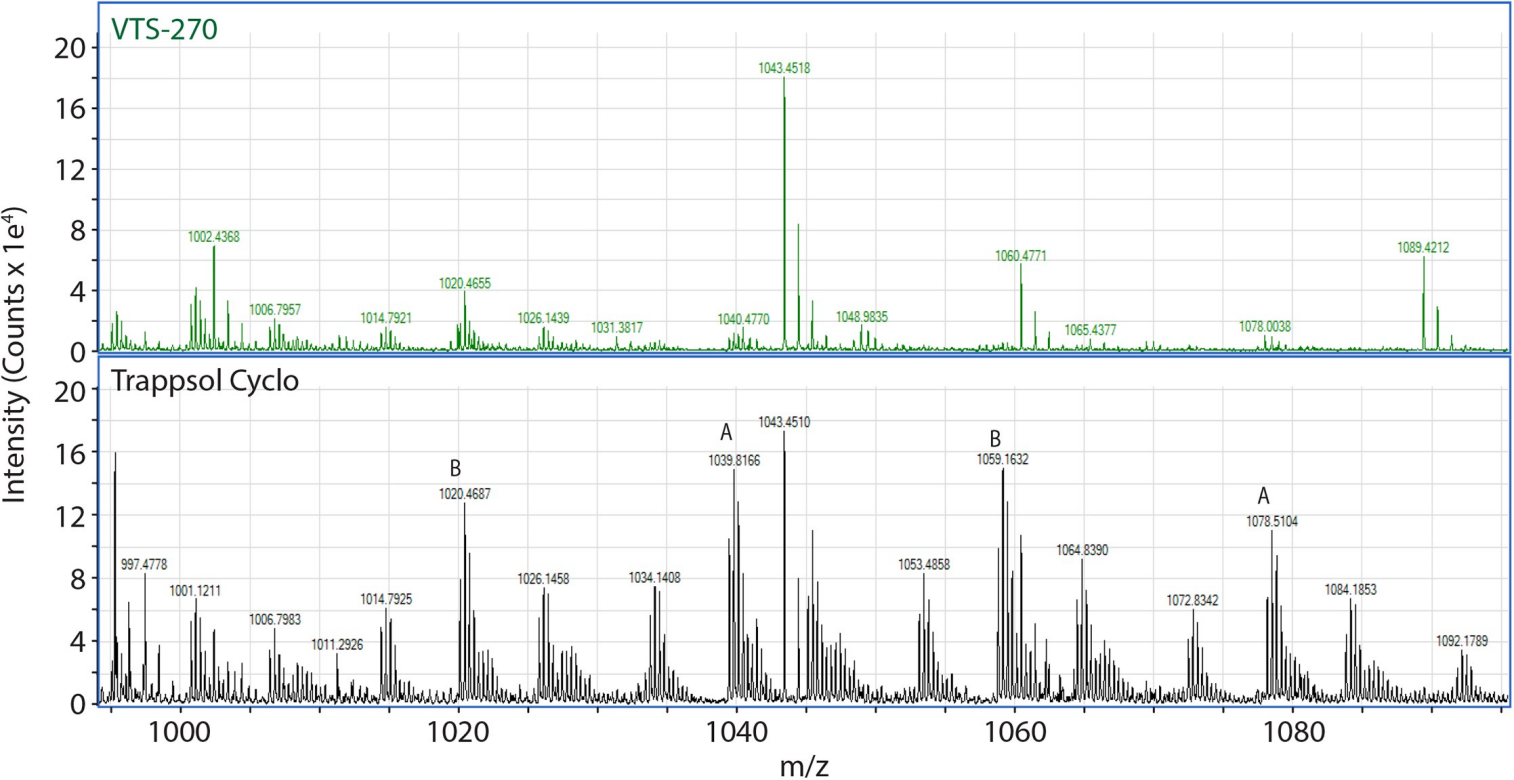
Mass spectra of a portion of the heat maps of VTS-270 and Trappsol Cyclo showing the m/z region dominated by triply charged dimeric ions. Ions A = [DS_n_ +2NH_4_ +H]^+3^ and Ions B = [DS_n_+DS_n-1_+2NH_4_+H]^+3^. See text for detailed description of the spectra. The spectra show clear differences with substantial intensity of triply charged dimers for Trappsol Cyclo but not for VTS-270.

Figs 2 and 3 represent a portion of the characteristic “fingerprint” of these molecular mixtures. Additional differences in the fingerprints are shown for VTS-270 and Trappsol Cyclo in Fig 4. This figure shows mass spectra from the region of Fig 2 over a mass range from ~990 to 1100 Da and drift times of ~25 to 27 msec. Here, compositional differences between the two materials are also readily apparent. Ions of the form of triply charged dimers of both homo and hetero origin (ie, [DS_n_ +2NH_4_ +H]^+3^ and [DS_n_+DS_n-1_+2NH_4_+H]^+3^; labeled A and B, respectively, in Fig 4) are visible in both panels but less intense in the VTS-270 sample than in the Trappsol Cyclo sample.

### Homodimer and heterodimer ion fractions

**Fig 5:**
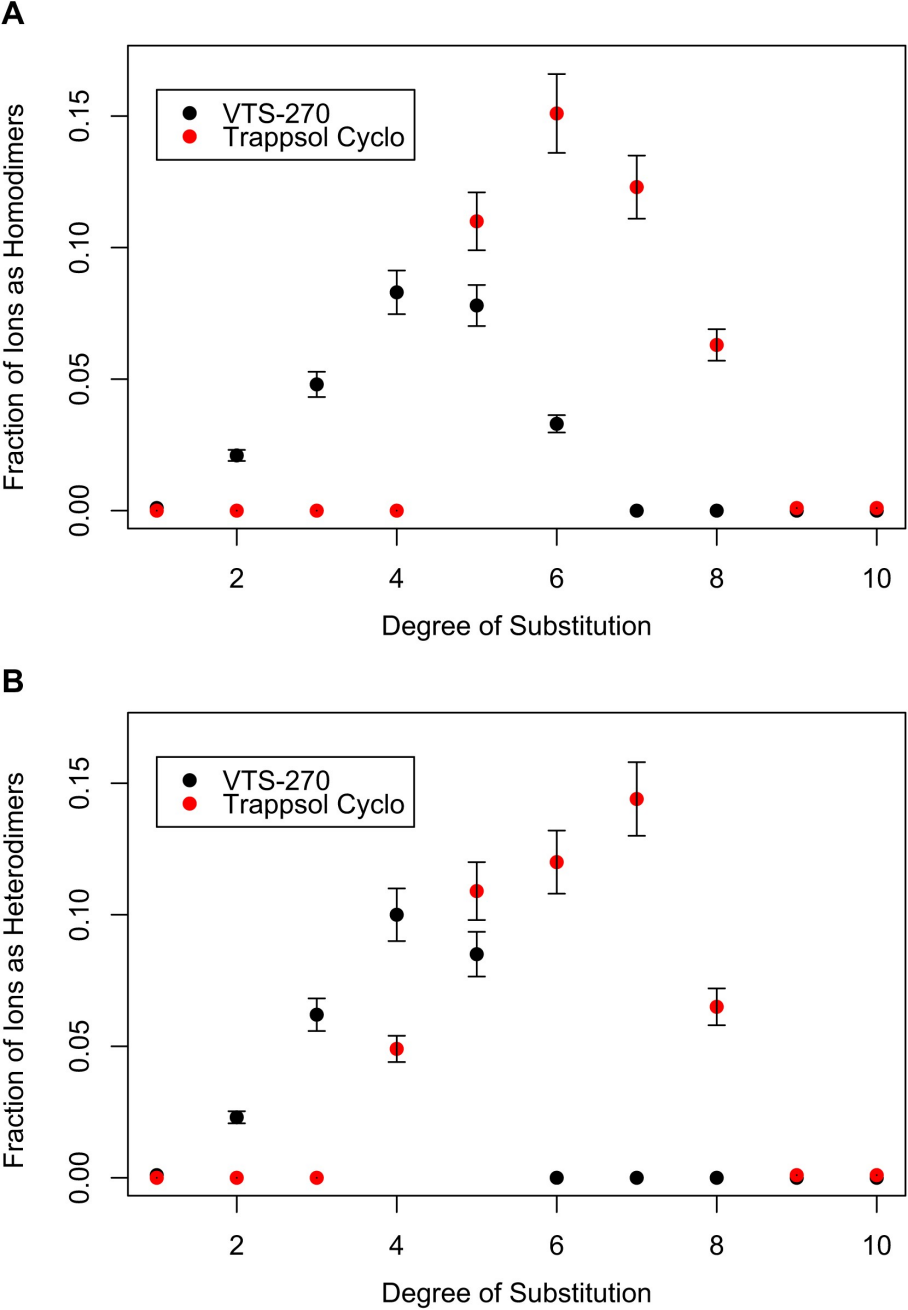
Fraction of dimers as a function of the degree of substitution. Fractional intensity of homodimer ions, relative to total hydroxypropyl-beta-cyclodextrin (HPβCD) ion signal, plotted as a function of the degree of substitution (A); fractional intensity of heterodimer ions, relative to total HPβCD ion signal, plotted as a function of the degree of substitution (B). The fractional intensity of ions as heterodimers and homodimers is greater with Trappsol Cyclo (red circles) than with VTS-270 (green triangles).

Variations in the relative intensities of dimeric ions are presented in Fig 5. The fraction of total ions represented by homodimers observed as a function of DS for both VTS-270 and Trappsol Cyclo are shown in Fig 5A. Similar fractional intensity changes as a function of DS are seen for both materials, but the relative fractional intensities of these dimers appear to be greater for Trappsol Cyclo than for VTS-270. Fig 5B shows the fraction of total ions represented by heterodimers as a function of DS for both materials. The difference between the two is more apparent in this case as the degree of heterodimer formation is seen to be much greater for Trappsol Cyclo than for VTS-270, particularly at higher DS. While these data were collected at 5 μM solution conditions, it is important to note that these observable differences were preserved under conditions of a 400-fold dilution in 80% ACN.

### Ion collision cross sections

Collision cross section (Ω) areas of the major ions of HPβCD were calculated using the VTS-270 and Trappsol Cyclo data as described and presented in Tables 1–6. These novel data were generated and included to add to the growing publicly available characterization of the ion collision cross sections measurements of various molecular species. While there are differences between VTS-270 and Trappsol Cyclo in the various ions present as described above, the ion collision cross sections for each of the ions present in both materials were similar, as expected.

**Table 1:** Ions as [M+H]^+^.

		VTS-270	Trappsol Cyclo
	m/z	Ω ± SD (Å^2^)	Ω ± SD (Å^2^)
**DS1**	1193.419	338.6 ± 0.3	-
**DS2**	1251.461	351.2 ± 0.5	-
**DS3**	1309.503	361.0 ± 0.2	-
**DS4**	1367.544	370.9 ± 0.5	-

**Table 2:** Ions as [M+NH_4_]^+^.

		VTS-270	Trappsol Cyclo
	m/z	Ω ± SD (Å^2^)	Ω ± SD (Å^2^)
**DS1**	1210.460	337.9 ± 0.2	-
**DS2**	1268.504	349.7 ± 0.6	350.5 ± 1.5
**DS3**	1326.546	361.9 ± 0.3	363.1 ± 0.4
**DS4**	1384.588	371.2 ± 0.3	371.5 ± 0.5
**DS5**	1442.630	377.8 ± 0.2	377.0 ± 0.4
**DS6**	1500.671	385.2 ± 0.2	384.4 ± 0.4
**DS7**	1558.697	-	394.4 ± 0.4
**DS8**	1616.738	-	403.0 ± 0.5
**DS9**	1674.780	-	410.5 ± 0.4

**Table 3:** Ions as [M_2_+H + NH_4_]^+2^.

		VTS-270	Trappsol Cyclo
	m/z	Ω ± SD (Å^2^)	Ω ± SD (Å^2^)
**DS1**	1201.932	-	-
**DS2**	1259.974	-	-
**DS3**	1318.016	521.2 ± 0.7	-
**DS4**	1376.058	539.1 ± 1.2	-
**DS5**	1434.010	555.1 ± 0.3	557.8 ± 1.3
**DS6**	1492.142	-	572.3 ± 0.5
**DS7**	1550.183	-	586.9 ± 0.6

**Table 4:** Ions as [M_2_ + 2NH_4_]^+2^.

		VTS-270	Trappsol Cyclo
	m/z	Ω ± SD (Å^2^)	Ω ± SD (Å^2^)
**DS1**	1210.460	-	-
**DS2**	1268.504	504.1 ± 0.8	-
**DS3**	1326.546	521.2 ± 0.5	-
**DS4**	1384.588	538.7 ± 0.4	539.6 ± 0.6
**DS5**	1442.630	555.5 ± 0.3	557.3 ± 0.2
**DS6**	1500.671	571.1 ± 0.6	573.3 ± 0.2
**DS7**	1558.697	-	587.8 ± 0.2
**DS8**	1616.738	-	601.5 ± 0.3

**Table 5:** Ions as [M_n_ + M_(n-1)_ + H+ NH_4_]^+2^.

		VTS-270	Trappsol Cyclo
	m/z	Ω ± SD (Å^2^)	Ω ± SD (Å^2^)
**DS1**	N/A	-	-
**DS2**	1230.953	-	-
**DS3**	1288.995	512.5 ± 0.7	-
**DS4**	1347.037	530.1 ± 1.1	-
**DS5**	1405.079	547.1 ± 0.5	549.6 ± 0.7
**DS6**	1463.121	563.2 ± 1.4	565.1 ± 0.6
**DS7**	1521.162	-	579.5 ± 0.4
**DS8**	1579.204	-	593.5 ± 0.1

**Table 6:** Ions as [M_n_ + M_(n-1)_ + 2NH_4_]^+2^.

		VTS-270	Trappsol Cyclo
	m/z	Ω ± SD (Å^2^)	Ω ± SD (Å^2^)
**DS1**	N/A	-	-
**DS2**	1239.466	-	-
**DS3**	1297.508	512.4 ± 0.3	-
**DS4**	1355.550	530.2 ± 0.5	-
**DS5**	1413.592	547.4 ± 0.4	549.4 ± 0.5
**DS6**	1471.634	563.9 ± 0.4	566.0 ± 0.1
**DS7**	1529.676	-	580.9 ± 0.1
**DS8**	1587.718	-	594.6 ± 0.1

Tables 1–6 show values of Ω for a series of different ions with varying DS observed in the m/z region between 1200 and 1620 Da. As seen in the mass spectra in Figs 3 and 4, these ions consist of a mixture of adducts of protons as singly and doubly charged species. As also seen in the spectra, not all ions of each adduct type are present in both VTS-270 and Trappsol Cyclo; however, when both species are present, the values calculated for their collision cross sections are in close agreement as noted in Tables 1–6.

## Discussion

Using electrospray coupled with ion mobility mass spectrometry, we demonstrated substantial differences between HPβCDs from two different sources currently being administered to NPC1 subjects. Specifically, both the DS and spectral fingerprints can be used to distinguish between HPβCDs of different sources; we found clear differences between two HPβCD products. Trappsol Cyclo was found to have a higher DS compared with VTS-270, increased levels of dimeric ions, and additional differences in ion mobility profiles. Furthermore, Trappsol Cyclo had greater nonspecific chemical noise with higher order complexes of HPβCDs compared with VTS-270.

The differences observed may be a consequence of the higher average DS of Trappsol Cyclo, giving rise to a greater propensity to form dimeric ions compared with VTS-270, which has a lower DS. For data acquisition, the instrument was operated under conditions designed to facilitate the observation of species existing in solution (ie, high temperature was not implemented). Accordingly, our observation of dimeric ions is most likely a reflection of species existing in solution prior to ion formation.

Dimer intensities appear to increase as a function of the DS, but absolute signal intensities arising from their presence is reduced by the decreasing intensity of the higher DS forms. In the case of Trappsol Cyclo, the overall higher DS leads to an overall relative increase in dimer signals. Considering the possibilities for hydrogen bonded non-covalent interactions that arise from greater levels of hydroxyl-propyl substitution, the existence of more extensive dimer interactions with Trappsol Cyclo rather than with VTS-270 is not surprising. The robustness of these interactions is apparently high because they were preserved during a 400-fold dilution and 80% ACN. Since these materials are normally administered at concentrations approximately 100-fold higher than used in these studies, the presence of dimers in solution appears to be likely. The observation of the strong heterodimers in Trappsol Cyclo suggests a different level of intermolecular interactions in Trappsol Cyclo than in VTS-270.

To our knowledge, there have not been any other studies directly comparing VTS-270 with Trappsol Cyclo regarding ion composition profiles or using the methods described herein to obtain and differentiate molecular fingerprints for the two materials. However, in a recently reported study that examined a series of α–, β– and γ–cyclodextrins for potential use in NPC, the average DS was noted to be 7.0 for Trappsol Cyclo and 4.3 for VTS-270, which was comparable with our findings, and the binding constants for unesterified cholesterol were found to be 3,250 ± 80 M^-1^ for Trappsol Cyclo and 4,400 ± 66 M^-1^ for VTS-270 [6].

Further studies are needed to examine potential differences in biological and therapeutic effects of Trappsol Cyclo and VTS-270. The data presented here strongly suggest that biological and potential therapeutic equivalence should not be assumed, as the activities of individual molecular species are not yet well understood or described. In the preparation of HPβCDs, the proportion of contaminants has been shown to be greater with HPβCDs with higher DS [15], and this holds true in comparing Trappsol Cyclo with VTS-270. The differences in the DS, dimer ion intensity, chemical noise, and contaminants indicate that VTS-270 and Trappsol Cyclo are not chemically equivalent and therefore may not be biochemically equivalent or lead to comparable formulations from a clinical development perspective. Therefore, when considering biological or clinical safety and efficacy data, it should not be assumed that effects observed with VTS-270 would also occur with Trappsol Cyclo and vice versa. Each product must be examined and evaluated independently and comparatively to fully understand the potential clinical similarities and differences with regard to safety and efficacy.

## Conclusions

These studies demonstrate the significant complexity that exists within the composition of HPβCDs. Based on ion mobility and mass spectral data, two HPβCD materials (VTS-270 and Trappsol Cyclo) were found to have substantial mass spectral differences and thus should not be considered to be chemically equivalent. Further research is needed to determine the impact these differences may have biologically and/or clinically in investigations of NPC.
