# Correction: Characterization of hydroxypropyl-beta-cyclodextrins used in the treatment of Niemann-Pick Disease type C1

**DOI:** 10.1371/journal.pone.0192424

**Published:** 2018-02-01

**Authors:** Alfred L. Yergey, Paul S. Blank, Stephanie M. Cologna, Peter S. Backlund, Forbes D. Porter, Allan J. Darling

The Competing Interest statement for this paper is incorrect. The correct statement is: Dr. Allan Darling was involved previously in additional research and testing on HPBCDs with a third party company. This related, unpublished work was performed under a commercial contract for which a confidentiality agreement prevents disclosure of the research details as well as the company and related product names. The authors confirm that this related HPBCD work by Dr. Darling was fully independent from the work published in the *PLOS ONE* article.

The Data Availability statement for this paper is incorrect. The correct statement is: All relevant data underlying this study are available from Zenodo (https://zenodo.org/record/573845#.Wh9DtkqnGUm).

The caption for Fig 5 is incorrect. Please see the complete, correct Fig 5 caption here.

**Fig 5:**
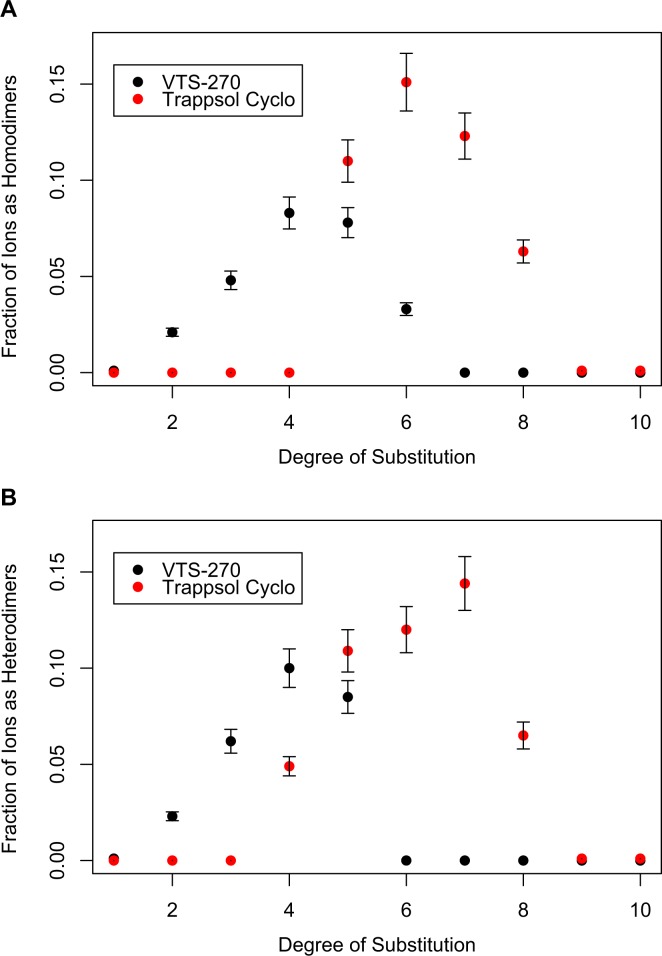
Fraction of dimers as a function of the degree of substitution. Fractional intensity of homodimer ions, relative to total hydroxypropyl-beta-cyclodextrin (HPβCD) ion signal, plotted as a function of the degree of substitution (A); fractional intensity of heterodimer ions, relative to total HPβCD ion signal, plotted as a function of the degree of substitution (B). The fractional intensity of ions as heterodimers and homodimers is greater with Trappsol Cyclo (red circles) than with VTS-270 (green triangles). The points represent the mean value ± standard deviation of 4 replicate measurements of each material.
